# Comprehending the cuproptosis and cancer-immunity cycle network: delving into the immune landscape and its predictive role in breast cancer immunotherapy responses and clinical endpoints

**DOI:** 10.3389/fimmu.2024.1344023

**Published:** 2024-01-19

**Authors:** Xiangwei Liu, Feng Xu, Kunkun Zhao, Yunfei Liu, Guolin Ye, Xin Zhang, Yanyu Qu

**Affiliations:** ^1^ Department of Breast Surgery, The First People’s Hospital of Foshan, Foshan, China; ^2^ Department of Anesthesiology, The First People’s Hospital of Foshan, Foshan, China; ^3^ Department of Breast Surgery, Foresea Life Insurance Guangzhou General Hospital, Guangzhou, China; ^4^ Department of General, Visceral, and Transplant Surgery, Ludwig-Maximilians-University Munich, Munich, Germany; ^5^ Department of Pathology, the Second People’s Hospital of Foshan, Foshan, China

**Keywords:** BC, cuproptosis, cancer-immunity cycle, immunotherapy response, prognosis

## Abstract

**Background:**

The role of cuproptosis, a phenomenon associated with tumor metabolism and immunological identification, remains underexplored, particularly in relation to the cancer-immunity cycle (CIC) network. This study aims to rigorously examine the impact of the cuproptosis-CIC nexus on immune reactions and prognostic outcomes in patients with breast cancer (BC), striving to establish a comprehensive prognostic model.

**Methods:**

In the study, we segregated data obtained from TCGA, GEO, and ICGC using CICs retrieved from the TIP database. We constructed a genetic prognostic framework using the LASSO-Cox model, followed by its validation through Cox proportional hazards regression. This framework’s validity was further confirmed with data from ICGC and GEO. Explorations of the tumor microenvironment were carried out through the application of ESTIMATE and CIBERSORT algorithms, as well as machine learning techniques, to identify potential treatment strategies. Single-cell sequencing methods were utilized to delineate the spatial distribution of key genes within the various cell types in the tumor milieu. To explore the critical role of the identified CICs, experiments were conducted focusing on cell survival and migration abilities.

**Results:**

In our research, we identified a set of 4 crucial cuproptosis-CICs that have a profound impact on patient longevity and their response to immunotherapy. By leveraging these identified CICs, we constructed a predictive model that efficiently estimates patient prognoses. Detailed analyses at the single-cell level showed that the significance of CICs. Experimental approaches, including CCK-8, Transwell, and wound healing assays, revealed that the protein HSPA9 restricts the growth and movement of breast cancer cells. Furthermore, our studies using immunofluorescence techniques demonstrated that suppressing HSPA9 leads to a notable increase in ceramide levels.

**Conclusion:**

This research outlines a network of cuproptosis-CICs and constructs a predictive nomogram. Our model holds great promise for healthcare professionals to personalize treatment approaches for individuals with breast cancer. The work provides insights into the complex relationship between the cuproptosis-CIC network and the cancer immune microenvironment, setting the stage for novel approaches to cancer immunotherapy. By focusing on the essential gene HSPA9 within the cancer-immunity cycle, this strategy has the potential to significantly improve the efficacy of treatments against breast cancer.

## 1 Introduction

Breast cancer, consistently identified as a leading cause of malignancy in women, has been witnessing an ongoing escalation in incidence rates annually (1). Each year, a staggering number of women are diagnosed with this disease, significantly impacting their lives and presenting formidable challenges to healthcare systems worldwide (2). Despite remarkable progress in early detection and therapeutic approaches in recent years, a substantial number of patients still face treatment hurdles and adverse prognoses (3). Conventional therapies, such as chemotherapy and radiotherapy, while effective in some cases, frequently lead to severe adverse effects, diminishing patients’ life quality. Furthermore, the inadequate response of certain subtypes of breast cancer to these traditional treatments highlights the constraints of existing medical approaches (4).

In the realm of breast cancer treatment, the past few years have witnessed the ascent of immunotherapy as a pivotal approach (5). This method harnesses the inherent strength of an individual’s immune response to target and destroy cancerous cells, marking a significant departure from conventional treatment strategies. Central to the efficacy of immunotherapy is the cancer-immunity cycle, a concept that depicts the dynamic interplay between the body’s immune defenses and cancerous cells (6). This cycle encompasses a multifaceted array of processes including the presentation of antigens, the activation of T-cells, and the subsequent eradication of tumor cells. These processes collectively function to bolster the immune system’s capacity to identify and eliminate cancer cells (7). In breast cancer research, significant advancements have been realized in elucidating and influencing the interplay between cancer and immunity, heralding new avenues for immunotherapeutic approaches (8). Despite achievements in treating various cancers, breast cancer’s response to immunotherapy is impeded by challenges, notably the differing immunological profiles of ‘hot’ and ‘cold’ tumor types and the intricate mechanisms of immune system evasion. Therefore, an enriched comprehension of the interplay between cancer and immunity in breast cancer emerges as a vital focus, which is the primary objective of this study.

Cuprotosis, also known as copper-induced cellular demise, represents a cellular biological phenomenon characterized by an aberrant elevation in intracellular copper ion levels, culminating in a process of programmed cell death (9). Copper is a pivotal trace element within living organisms, that plays multifaceted roles in various biological functions encompassing redox reactions, cellular signal transduction, and enzymatic activities (10). However, the excessive accumulation or anomalous distribution of copper can potentially trigger cuproptosis, a process of paramount significance for cellular viability. Scientific investigations have elucidated the close association between cuproptosis and the onset and progression of tumors, as copper assumes a pivotal role in governing tumor cell proliferation, invasiveness, and drug resistance (11). Furthermore, cuproptosis is intricately linked to the functioning of the immune system, given copper’s vital contributions to immune cell activity and antioxidant defense (12). A profound exploration of the mechanisms and regulation of cuproptosis promises to enhance our comprehension of its roles in tumorigenesis and immune modulation, thereby furnishing crucial insights for the development of novel anti-tumor therapeutic strategies. Consequently, cuproptosis, as a biological phenomenon, holds substantial research value and clinical application prospects with regards to its definition, biological functions, and its close interrelation with tumors and the immune system.

In recent years, bioinformatics has been widely employed in the diagnosis of numerous diseases (13, 14), and the prediction and treatment of immunity in a multitude of medical scenarios. The aim of our research was to harness the capabilities of the cuproptosis-cancer-immunity cycle (CIC) network in forging a comprehensive prognostic framework. This framework, designed to evaluate survival threats in breast cancer sufferers, amalgamates clinical data to formulate a nomogram, thereby augmenting its clinical relevance. Such enhancement assists medical professionals in formulating well-informed treatment strategies. Our results reveal a substantial link between the cuproptosis-CIC nexus and the response to immunotherapy in breast cancer cases, facilitating precise prognostication of survival challenges for these patients. Additional studies have illuminated the role of CIC genes in the cellular dynamics of breast cancer, positing the cuproptosis-CIC constellation as a viable biomarker for breast cancer therapy and management.

## 2 Materials and methods

### 2.1 Data acquisition

To examine the transcriptome profiles of TCGA-BRCA (breast cancer), we accessed clinical details and transcriptomic data from the GDC database. The dataset comprises a total of, 1222 transcriptome data samples, coupled with clinical records. This dataset encompasses 113 normal samples and, 1109 breast cancer tumor samples. After excluding samples with incomplete clinical information and normal samples, a final count of, 1091 breast cancer patient samples remained available for subsequent analyses. Among these, there are 327 samples from the breast cancer patient cohort, with an additional 50 samples sourced from the Korean breast cancer patient cohort, downloaded from GSE20685 and ICGC-BRCA, respectively. Following the retrieval, we employed thorough preprocessing methods to ensure the data’s analytical veracity. These steps included the normalization of gene expression levels, addressing missing data entries, and organizing the data based on gene symbols. For the integration of these datasets and the reduction of bias, the “sva” R package was utilized (15, 16). Differential expression patterns among genes were analyzed using the “limma” software package (17, 18).

### 2.2 Consensus clustering

In conducting Consensus Clustering, the R package ‘ConsensusClusterPlus’ was employed (19). This approach resulted in the bifurcation of the BC cohort into three distinct groupings (k = 3). To further confirm the steadfastness of these groupings, we applied the UMAP and tSNE methods (20, 21), utilizing the ‘ggplot2’ package in R (22).

### 2.3 Analysis utilizing LASSO regression

In our study, we initiated a preliminary examination to identify potential prognostic indicators. This was achieved through the application of univariate Cox regression analysis (23). The procedure entailed a thorough examination of each factor to determine its substantial influence on the survival outcomes, with statistical significance set at *P* < 0.05. The scope of our analysis covered 46 constituents involved in the cancer-immunity cycle (CICs), which are crucial for the survival prognosis in breast cancer (BC) patients. Further, we employed the LASSO (Least Absolute Shrinkage and Selection Operator) regression as a regularization strategy to avert overfitting and aid in identifying relevant features (24–26). This approach is instrumental in reducing the impact of less pertinent features by shrinking their coefficients to zero, thus streamlining the process of selecting a critical subset of features for further analysis. The implementation of LASSO regression was conducted through the ‘glmnet’ package in the R programming environment, where the most suitable λ parameter was ascertained through a process of tenfold cross-validation. In the final stages of this investigation, we pinpointed ten crucial genes through a complex Cox regression analysis. This approach involved selecting optimal lambda values and coefficients to construct a risk signature encompassing ten cancer-immune cycle (4-CIC) elements. In the end, four cuproptosis-CICs genes were identified for the construction of the prognostic model. Utilizing their individual gene expression profiles, we calculated a prognostic risk score for every patient within the cohort using the following formula: Risk score = e^(Exp. HSPA9 * 0.3731 - Exp. HSPA2 * 0.2042 - Exp. MICB * 0.1437 - Exp. CXCL13 * 0.0751).

### 2.4 Assessment of immune cell infiltration

In our study, we adopted a dual-faceted approach to evaluate immune cell presence in the specimens. This approach incorporated the use of both “CIBERSORT” and “ssGSEA” algorithms through R-based scripts (27, 28). The application of the CIBERSORT algorithm was crucial for determining the level of immune cells in the tumor tissues (23). In parallel, we used the “Estimate” package for estimating the proportions of stromal and immune cells within the tumor specimens.

### 2.5 DCA curve

The nomograms’ clinical efficacy is scrutinized using Decision Curve Analysis (DCA). By evaluating net gains at differing probability thresholds, this technique aids in balancing the danger of unnecessary medical interventions against the risk of failing to identify treatable illnesses. DCA presents an intricate schema for assessing the nomograms’ practicality in a clinical context, affirming their utility in shaping decision-making for treatments (29, 30).

### 2.6 *In vitro* cell culture

Acquired through ATCC, the cell lineage Hs 578T BC was cultivated in DMEM (sourced from Solarbio), supplemented with a 10% concentration of fetal bovine serum (provided by Solarbio, catalog number S9020) (31).

### 2.7 Gene silencing via siRNA interference

The Hs 578T cells were treated with a transfection process that involved the application of small interfering RNA (siRNA) directed at the HSPA9 gene, which can be identified by its catalog number sc-35520 from Santa Cruz. As a baseline for comparison, a control experiment was conducted using a non-specific siRNA, cataloged as sc-37007 from Santa Cruz. The transfection in these experiments was facilitated using Lipofectamine, 3000.

### 2.8 Investigation of Hs 578T cells proliferation via targeting HSPA9

We scrutinized the resilience of Hs 578T cellular configurations following siRNA exposure over a series of time intervals - namely, 0, 12, 24, 36, 48, and 72 hours. Simultaneously, an evaluation of the Hs 578T cells’ survival was performed using the Cell Count Kit-8 (Solarbio) method. This procedure entailed adding 10 μl of the CCK8 solution to each well, which was then subjected to a two-hour incubation at a stable environment of 37°C and a 5% CO2 atmosphere. Subsequent to the incubation, absorbance measurements at a 450 nm wavelength were precisely taken and recorded for further analysis.

### 2.9 Cell migration ability

To investigate the impact of HSPA9 on the migration capability of Hs 578T cells, we conducted both Transwell and wound-healing assays. The specific procedures were as follows: Hs 578T cells were separately transfected with control siRNA and siRNA targeting HSPA9. After 48 hours, cells from both the control group and the experimental group were collected for the Transwell assay. In the Transwell assay, 25,000 Hs 578T cells were resuspended in culture medium without FBS and then placed in the upper chamber of the Transwell insert, while the lower chamber was filled with culture medium containing 20% FBS. Cell fixation and crystal violet staining were performed at 24-hour and 36-hour time intervals, each for 50 minutes. Subsequently, photographs were taken, and statistical analysis was conducted.

Simultaneously, 300,000 Hs 578T cells were seeded in a 6-well plate, and a sterile needle was used to create a linear scratch between the cells, generating a gap. Photographs were taken at the 0-hour time point to document the initial cell gap distance. After 48 hours, photographs were taken again to record the status of cell migration. Cell migration distances were calculated using ImageJ, followed by statistical analysis and graphical representation using Prism 8.0.

### 2.10 Immunofluorescence staining of ceramide

To perform immunofluorescence staining of ceramide protein in Hs 578T cells, brief steps are shown as follows: Initially, cells must be cultured on glass coverslips. Subsequently, cells are fixed using either 100% methanol at room temperature for 5 minutes or 4% paraformaldehyde in phosphate-buffered saline (PBS) pH 7.4 for 10 minutes at room temperature. Post-fixation, the cells are to be washed thrice with ice-cold PBS. Depending on the primary antibody used, antigen retrieval may be required. This involves heating the cells in antigen retrieval buffer at 95°C for 10 minutes. If the target protein is intracellular, cell permeabilization is achieved using PBS containing 0.1–0.25% Triton X-100 for 10 minutes. The concentration of Triton X-100 must be optimized for the specific protein of interest. For blocking non-specific binding, cells are incubated in a blocking solution containing 1% BSA or serum in PBST (PBS + 0.1% Tween 20) for 30 minutes. Immunostaining involves incubating the cells with the primary ceramide antibody diluted in 1% BSA in PBST for 1 hour at room temperature or overnight at 4°C, followed by washing with PBS and incubation with the secondary antibody for 1 hour at room temperature in the dark. After secondary antibody incubation, cells are washed three times with PBS in the dark. Finally, the coverslip is mounted using a mounting medium and sealed with nail polish. The slides are stored in the dark at -20°C or +4°C until ready for microscopic examination.

### 2.11 TISCH database and best database

The TISCH database (32), a web-based repository of paramount significance, chiefly catalogues single-cell RNA-Seq data pertinent to the tumor microenvironment (TME). Leveraging this resource, we investigated the ubiquity of 6-CICs within a myriad of cell types present in BC. The single-cell TIME (scTIME) portal, accessible at http://scTIME.sklehabc.com, offers specialized analysis modules tailored for scTIME data, along with a unified cell annotation system. Apart from the evaluation of immune cell compositions and correlation analysis using precise cell type classifications, the portal also facilitates investigations into cell-to-cell interactions and the analysis of gene signatures specific to distinct cell types. Through single-cell analysis, we have gained insights into the communication networks among various cell types within the context of breast cancer patients. Additionally, the ‘BEST’ analytical model, accessible via ‘rookieutopia.com’, was utilized for examining cancer biomarkers and prognosticating immunotherapeutic outcomes. This methodology permitted a thorough examination of distinct risk stratified subgroups.

### 2.12 Analysis of statistical data

The evaluation of statistical data was executed using R software (version 4.1.3). Complementary analysis was facilitated through GraphPad Prism (GraphPad Software, USA). The benchmark for deeming statistical significance was established at *P* < 0.05. Symbols used in the representation are: * indicating *P* < 0.05, ** denoting *P* < 0.01, and *** for *P* < 0.001.

## 3 Results

### 3.1 Prognostic implications of CICs

**Figure 1:**
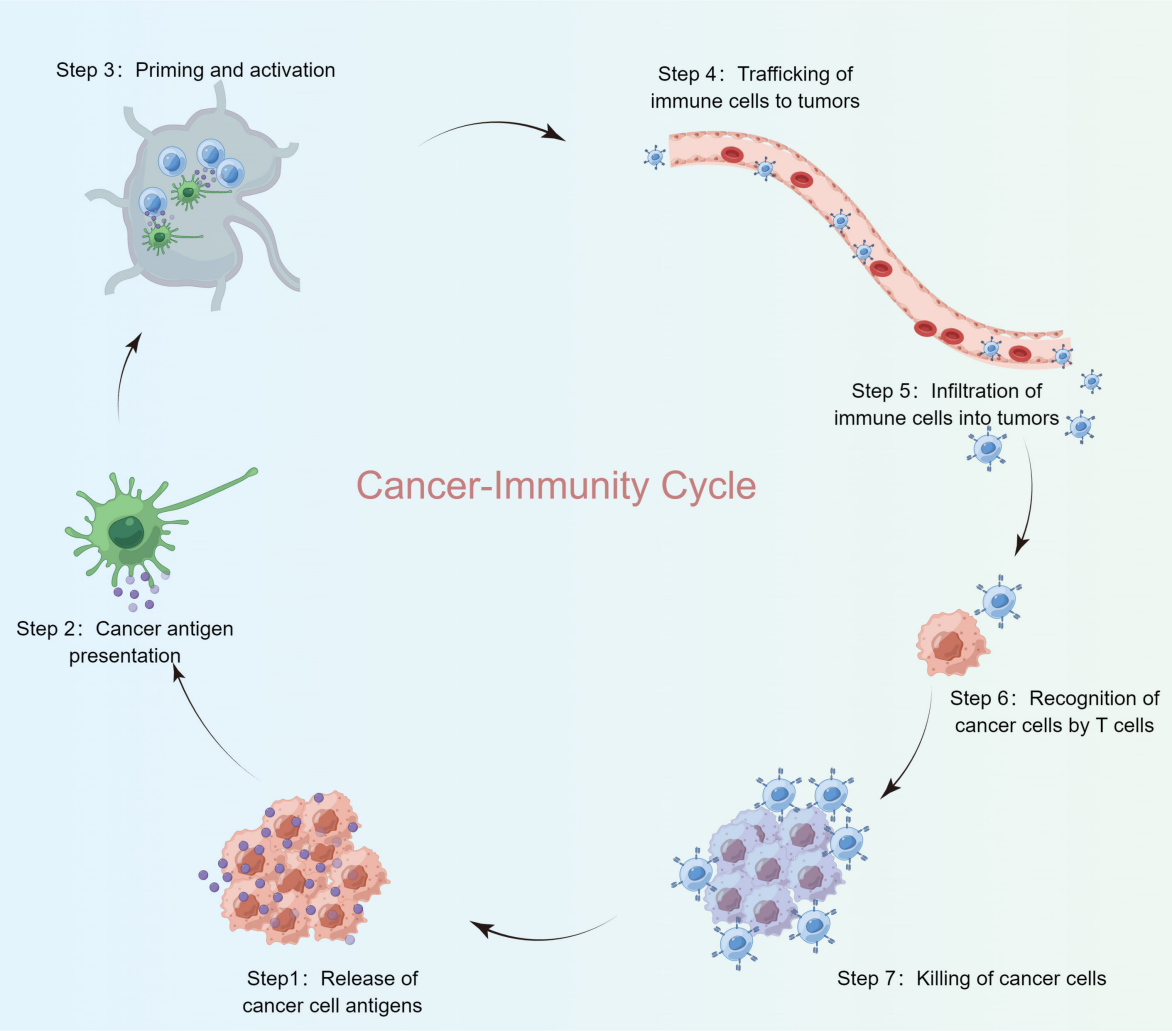
Cancer-immunity cycle steps.

The suppression of tumor growth via immune response follows a multi-step process, frequently referred to as the Cancer-Immunity Cycle. In this study, the landscape of immune responses against cancer was charted across seven distinct phases of this cycle (**Figure 1**). The analysis incorporated TCGA-BRCA data and 120 Cancer-Immunity Cycle components (CICs) from the TIP database.

**Figure 2:**
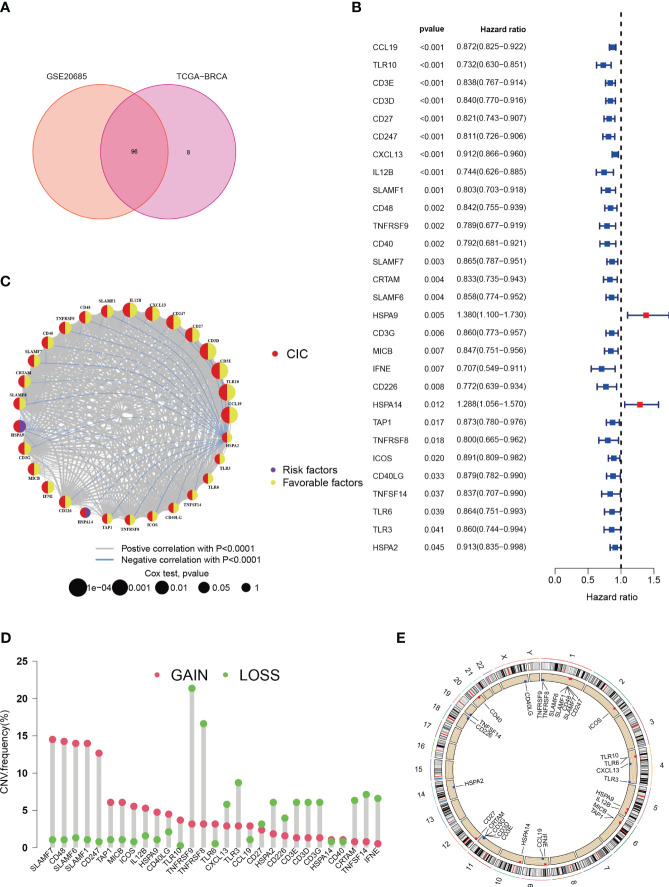
Characteristics of cancer-immunity cycle-associated genes in Breast cancer. **(A)** Venn plot for shared CICs in TCGA-BRCA and GSE20685 cohorts. **(B)** An association between survival outcomes and the 29 cuproptosis-CICs (*P* < 0.05). **(C)** Network among 29 cuproptosis-CICs (P<0.01). **(D)** In the TCGA-BRCA dataset, a study of 30 CICs revealed distinct CNVs. **(E)** The chromosomal positioning of these CICs is delineated.

In the LIHC-BRCA cohort, there are records of expression data for 104 CICs, while the GSE20685 dataset encompasses expression profiles for 96 CICs (**Figure 2A**).Through uniCox analysis, prognostically significant CICs were selected. Notably, 29 CICs displayed a Kaplan-Meier estimate under 0.05, followed with a P-value lower than 0.05 (**Figure 2B**). Among these, 2 CICs were linked to unfavorable breast cancer prognoses, whereas 27 suggested better outcomes. A network diagram was generated to display the relationships between these CICs, highlighting 29 interrelated CIC genes (**Figure 2C**). Additionally, an examination of Copy Number Variations (CNVs) from TCGA data was conducted to investigate chromosomal aberrations typical in cancerous growths (33). This entailed mapping the chromosomal locations of each CIC and quantifying their variations (**Figures 2D, E**). Significant chromosomal alterations were observed, particularly ‘gains’ and ‘losses’ on chromosome 1, involving SLAMF7, and on chromosome 1 concerning TNFRSF9.

### 3.2 Consistent clustering

**Figure 3:**
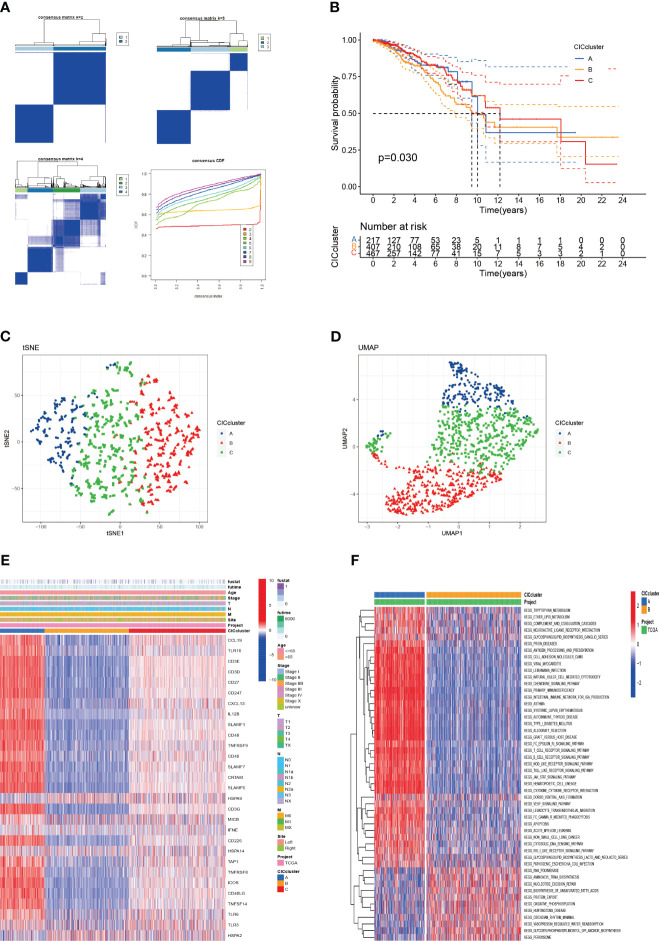
The categorization of breast cancer variants through cuproptosis-CICs delineation is presented. **(A)** The acceptance of a consensus matrix for k =3 was executed via consensus clustering. **(B)** The survival likelihood for three distinct subtypes demonstrated statistical significance (P < 0.05). **(C, D)** Through UMAP analysis, three subtypes were distinctly categorized based on the expression levels of CICs. **(E)** An alternative analysis of Clusters A and B was conducted using GSVA, focusing on the enrichment of KEGG pathways. **(F)** The clinical and pathological characteristics were outlined for the three CICs expression subtypes.

To deepen the understanding of 29 pivotal genes in breast cancer, we applied consensus clustering via the “ConsensusClusterPlus” package within the R environment. This approach delineated a novel subgrouping within the study population, creating three distinct clusters (**Figure 3A**). Kaplan-Meier survival graphs revealed striking survival rate contrasts among these clusters (*P* < 0.01), as illustrated in **Figure 3B**. The application of Uniform Manifold Approximation and Projection (UMAP) analysis lent support to the precision of these classifications (**Figures 3C, D**). Additionally, our investigation probed the link between the expression of 29 cancer immunity-related genes and clinicopathological traits across these clusters. The heatmap showed significant differential expression of genes such as HSPA9, CCL19, and CD247 in cluster B, which may implicate their roles in tumor progression (**Figure 3E**). To explore the distinct expression patterns of these 29 genes across subtypes, we conducted a Gene Set Variation Analysis (GSVA) to compare KEGG pathway enrichment between clusters A and B. This analysis unveiled significant discrepancies in survival outcomes, mirroring the findings depicted in **Figure 3B** (**Figure 3F**). Cluster B, which is associated with an unfavorable prognosis, predominantly exhibited pathways associated with T cell and B cell receptor signaling, antigen processing, NK cell-mediated cytotoxicity, primary immunodeficiency, and other pathways implicated in oncogenesis. These observations provide potential insights into the survival disparities observed. It is noteworthy that, aside from the suppression of immune processes, pathways related to apoptosis, crucial for high-risk breast cancer patients, were also hindered. This inhibition is expected to promote tumor cell proliferation and resistance to immune-based therapeutic agents.

### 3.3 Characterization of immune penetration in distinct subtypes

**Figure 4:**
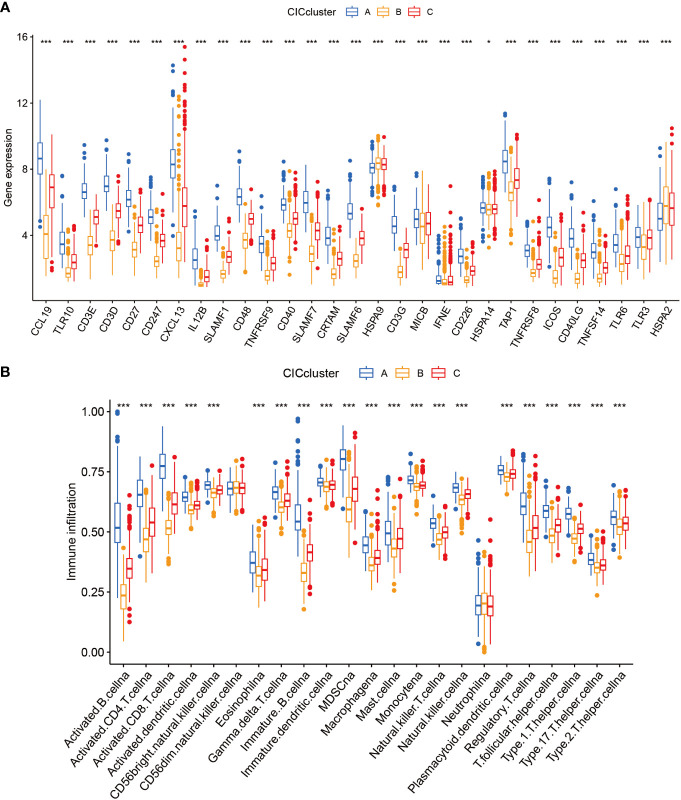
Examination of 3 clusters based on cuproptosis-CICs delineates distinct immune infiltration profiles and gene expression patterns. **(A)** Depicts the expression levels of cuproptosis-CICs in three different subtypes. **(B)** Illustrates the diverse patterns of immune infiltration associated with these three subtypes. (Statistical significance determined by Wilcox test: *P < 0.05, ***P < 0.001).

In this segment, we articulate the heterogeneous expression profiles of 29 distinct cancer-immune constructs (CICs) discerned within three separate subgroups. A closer scrutiny of **Figure 4A** underscores an enhanced expression of CICs, especially within subgroup B. It’s essential to acknowledge the findings from prior investigations which associate the upregulation of HSPA9 gene expression with improved clinical prognoses. This observation leads us to conjecture the central role of HSPA9 as a gene linked with sphingolipids, crucial in the body’s primary immune defense against the proliferation of tumors. With subgroup B linked to unfavorable clinical outcomes, it’s plausible that these CICs play a pivotal role in the progression of breast cancer (BC), potentially offering new avenues for targeted therapeutic interventions in BC. Moreover, there are discernible differences in immune cell infiltration among the subtypes (**Figure 4B**). The decreased presence of immune cells in subgroup B, including activated CD8 T cells and CD4 T cells, might indicate a role for CICs in promoting an immunosuppressive environment within tumors.

### 3.4 Construct CICs predictive model

**Figure 5:**
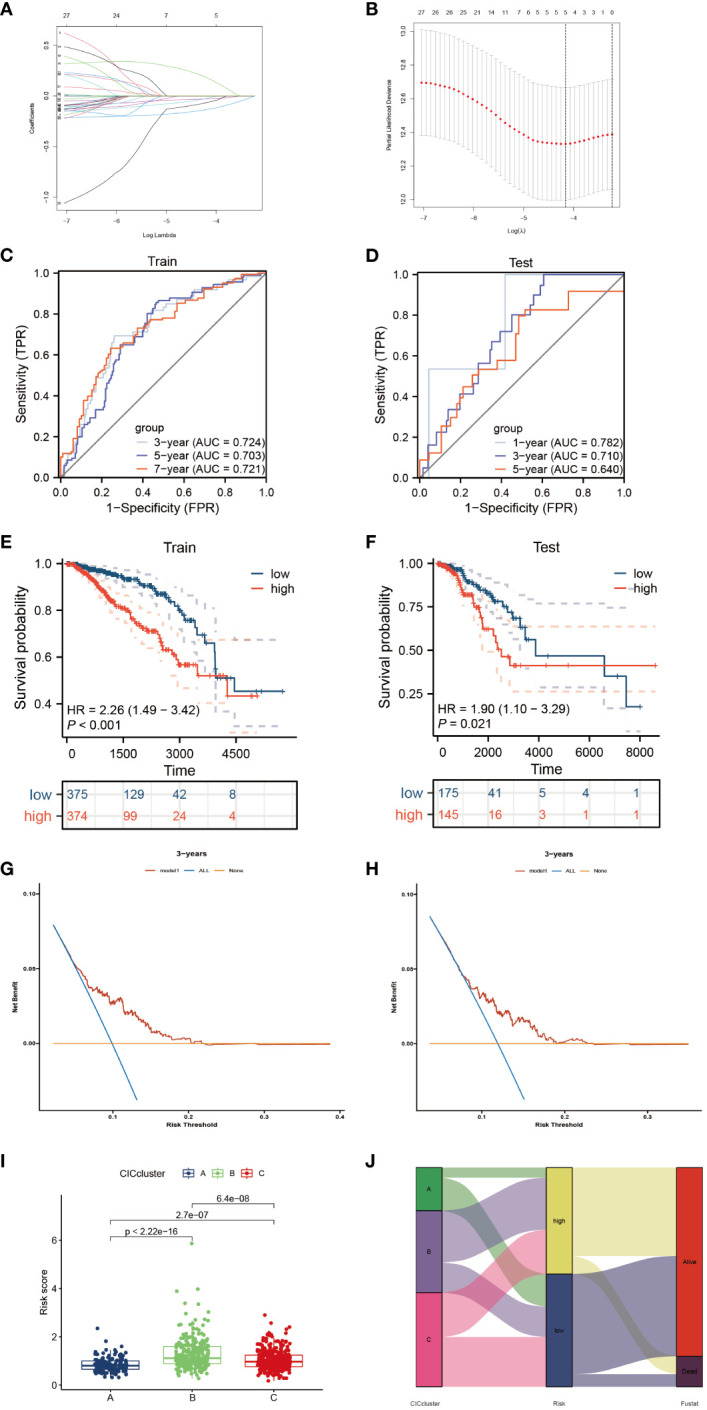
Elucidation of a signature linked to the cancer-immunity cycle’s predictive capacity. **(A, B)** Utilizing LASSO methodology, a prognostic ensemble of 4 cuproptosis-CICs was delineated. **(C, D)** Assessment of the risk score model’s efficacy through time-dependent ROC, focusing on its sensitivity and specificity parameters. **(E, F)** Analysis of survival probabilities across distinct risk stratified groups. **(G, H)** Applying decision curve methodology to evaluate a 4 cuproptosis-CICs risk score model in forecasting survival outcomes. **(I)** Compilation of risk scores within three distinct CIC clusters. **(J)** A comprehensive alluvial chart depicting the relationship between CIC cluster categorization and subsequent survival status.

**Figure 6:**
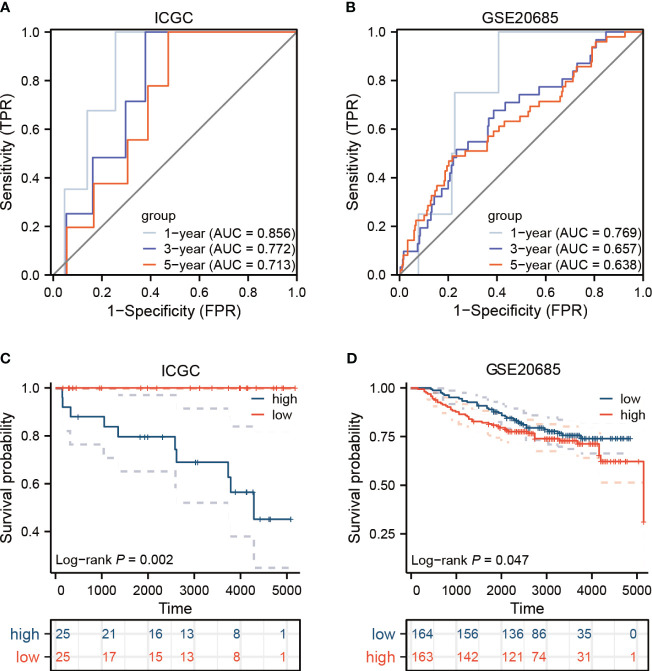
In the external validation cohorts, the model’s efficacy is assessed. **(A, B)** Displayed here are the ROC curves for two distinct breast cancer cohorts. **(C, D)** The survival outcomes for the ICGC and GEO groups are presented.

To devise a model capable of forecasting the risk profile for each patient, our strategy entailed dividing the “TCGA-BRCA” cohort into two unique sections: one for training purposes and another for validation. The LASSO-Cox regression technique was applied to scrutinize genes exhibiting varied expression in the training section, culminating in the isolation of 4 critical genes connected to risk (**Figures 5A, B**). We coined the term “riskscore” for this calculated risk metric, which is elaborated in **Supplementary Table 1** with respective coefficients for each gene identified. Time-dependent ROC curve analysis, targeting 1, 3, 5 and 7-year overall survival predictions in both the training/testing groups and external validation cohorts (ICGC, GSE20685), revealed our model’s strong prognostic abilities for overall survival (**Figures 5C, D**; **6A, B**). Importantly, a significant survival advantage was observable in the low-risk group compared to their high-risk peers (*P* < 0.05) (**Figures 5E, F**; **6C, D**). The decision curve analysis (DCA) corroborated the clinical relevance of our model, indicating potential improvements in survival rates for patients with breast cancer (**Figures 5G, H**). Risk scores varied considerably, correlating with three subtypes associated with the signature genes (**Figures 5I, J**). Furthermore, we utilized an alluvial diagram to clarify the interplay between cluster groupings, riskscore, and survival status.

### 3.5 Investigate CICs expression via single-cell analysis

**Figure 7:**
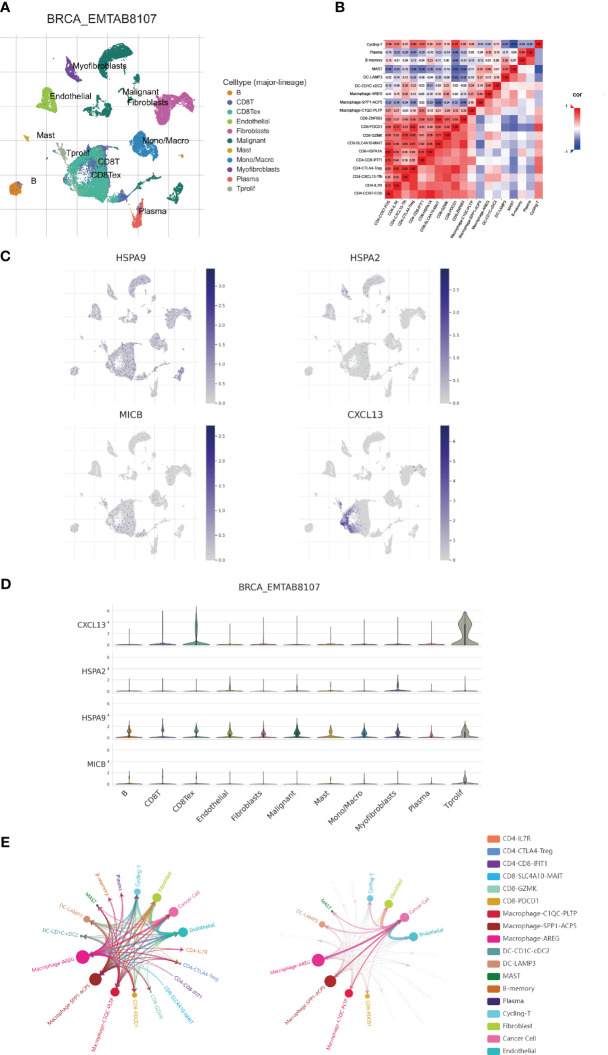
Examination of expression patterns in 4 cuproptosis-CICs on a single-cell level. **(A)** Annotation of nine distinct cellular phenotypes. **(B)** Elucidation of the interactions among immune cells within the context of breast carcinoma, utilizing single-cell methodologies. **(C, D)** Assessment of 4 cuproptosis-CICs expression magnitudes within breast carcinoma tissues. **(E)** Analysis of intercellular communication dynamics involving neoplastic cells in breast cancer scenarios.

In this progressed segment of our study, we applied an approach focusing on single-cell analysis to investigate the expression patterns of CIC in immune cells from patients with breast cancer. We identified and classified nine unique cellular types (**Figure 7A**), and charted the expression levels of six specific CICs. These CICs demonstrated increased expression in these cell varieties, as evidenced by data extracted from the TISCH and scTIME databases (**Figures 7B–D**). In line with previous studies, the HSPA9 indicator was mainly observed in tumor cells, while HSPA9 was predominantly associated with malignant cells. This information is instrumental for guiding the formulation of gene therapies that target specific cell types. Furthermore, our research delved into the dynamics of tumor-immune cell interactions. Through single-cell analysis, we have gained insights into the communication networks among various cell types within the breast cancer patients’ microenvironment. It is noteworthy that within the breast cancer tumor microenvironment, multiple subtypes of macrophages exhibit extensive interactions with breast cancer cells. Simultaneously, they also engage in communication with CD8T cells expressing PDCD1 (**Figure 7E**).

### 3.6 Immunotherapy response and immune infiltration

**Figure 8:**
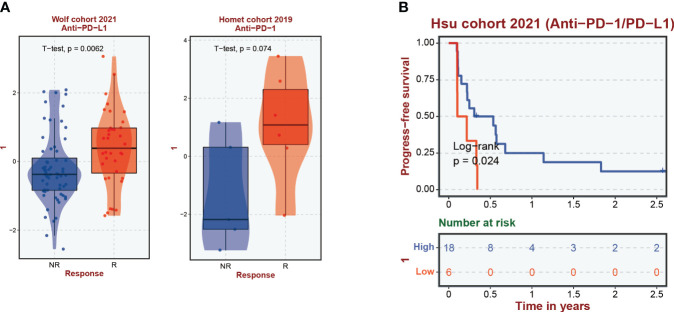
Correlation of cuproptosis-CICs expression with immune response efficacy and patient survival in breast cancer. **(A)** Demonstrates the levels of cuproptosis-CICs expression within breast cancer patients experiencing immune responses. **(B)** Explores the association between cuproptosis-CICs expression levels and the survival rates of individuals diagnosed with breast cancer.

**Figure 9:**
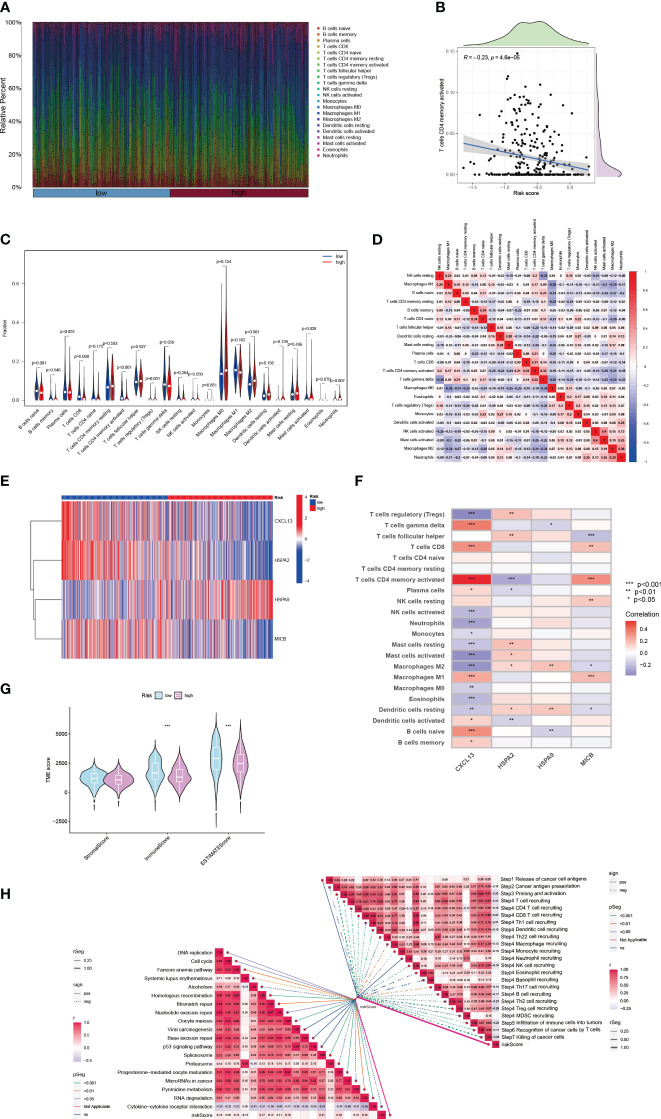
The immune landscape of breast cancer characterized by varying risk scores. **(A)** Associations between risk scores and diverse immune cell infiltration levels. **(B)** Analysis of the relationship between risk scores and the presence of active CD4 memory T cells in breast cancer tissue. **(C)** Immune cell composition differences in groups with high and low risk. **(D)** Interrelations among different immune cells. **(E)** In breast cancer patients of distinct risk levels, the expression profiles of 4 cuproptosis-CICs are examined. **(F)** Investigations into the interplay between immune cells and 4 cuproptosis-CICs. **(G)** Approximated scores for different risk subgroups. **(H)** Studies on the connection between risk scores and both the cancer-immunity cycle and functional pathways. (*P < 0.05, **P < 0.01, ***P < 0.001).

A comprehensive analysis has underscored an intensified expression of PDCD1, particularly within the subgroup B cluster. Subsequent inferences suggest a potential for increased immunotherapy efficacy in breast cancer cases where CIC expression is elevated. Data exploration through the BEST database highlighted a pattern: breast cancer patients responding favorably to immunotherapy treatments often display a surge in CIC expression (**Figure 8A**). Moreover, a correlation has been established between high CIC expression in breast cancer patients and a notable improvement in overall survival rates post-immunotherapy, substantiated by statistical data (*P* < 0.05) (**Figure 8B**). The study of the tumor microenvironment, with a specific emphasis on the role of the immune system, reveals its critical impact on tumor evolution. Immune system dysfunction is implicated in enabling tumor cells to evade detection (23, 34). To evaluate the microenvironmental composition in individuals at varying levels of breast cancer risk, we utilized the CIBERSORT R algorithm. Initially, we assigned risk scores to the patients and employed them to determine the relative abundance of a wide array of immune cells in each case (**Figure 9A**). It is noteworthy that a negative correlation with a coefficient of -0.23 was observed between the presence of CD4 memory T cells and the patients’ risk scores (**Figure 9B**). Within breast cancer tissues, we identified a predominant presence of macrophages M2, neutrophils and activated Mast cells (**Figure 9C**). This finding suggests their significant involvement in the progression of breast cancer. Furthermore, a notable correlation with a coefficient of 0.39 was uncovered between macrophages M2 and neutrophils in the microenvironment of breast cancer (**Figure 9D**). The risk assessment model was developed based on a signature comprising 4 distinct CICs, each demonstrating a wide range of expression patterns and exhibiting a robust connection with diverse immune cell infiltrates. Specifically, there was a positive correlation observed between macrophages M2 and HSPA9 expression (**Figures 9E, F**). Further analysis encompassed the computation of stromal, immune, and tumor scores for patients across different risk strata. This analysis was conducted using the “estimate” R package, which allowed for the examination of expression profiles (**Figure 9G**). Notably, individuals categorized as high-risk displayed a compromised immune response, accompanied by heightened activity in spliceosome and RNA degradation pathways (**Figure 9H**).

### 3.7 Nomogram predicts BC patients survival

**Figure 10:**
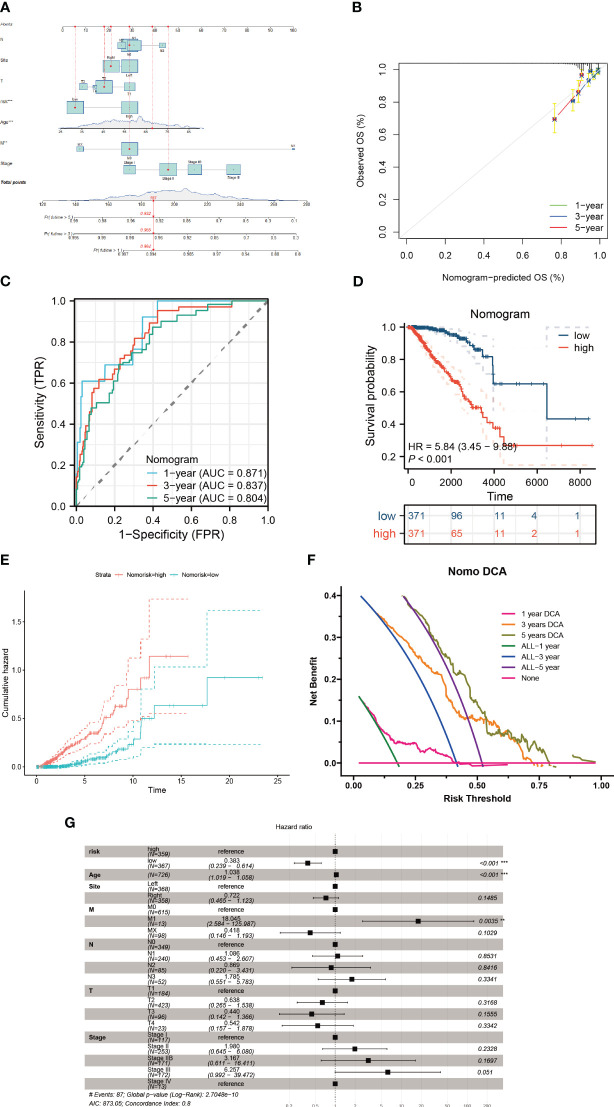
Construction of a nomogram model. **(A)** The nomogram is formulated integrating risk scores with clinicopathological characteristics. **(B)** A calibration graph substantiates the nomogram’s accuracy. **(C, D)** Discrepancies in survival among breast cancer patients evaluated using the nomogram scoring. **(E)** The cumulative hazard curve delineates survival probabilities over a timeline. **(F)** Decision Curve Analysis (DCA) of the nomogram illustrating the survival outcomes in breast cancer patients. **(G)** Multifactorial Cox regression assessment of clinical attributes and risk scores in these patients **P < 0.01; ***P < 0.001.

To assess the influence of various clinical parameters, such as tumor location, patients’ ages, and disease stage, on tumor progression, we developed a nomogram that integrates these factors with the risk score (**Figure 10A**). The accuracy of this nomogram was evaluated using a calibration plot (**Figure 10B**) and a series of cumulative risk graphs, demonstrating a direct association between higher scores and increased survival risk among breast cancer patients (**Figures 10C–E**). Utilizing decision curve analysis, we observed significant long-term benefits associated with the use of this nomogram in clinical decision-making for breast cancer patients (**Figure 10F**). Furthermore, a forest plot analysis identified age, M1 stage and the risk score as critical determinants of the nomogram’s effectiveness (**Figure 10G**).

### 3.8 Cuproptosis-CICs network

**Figure 11:**
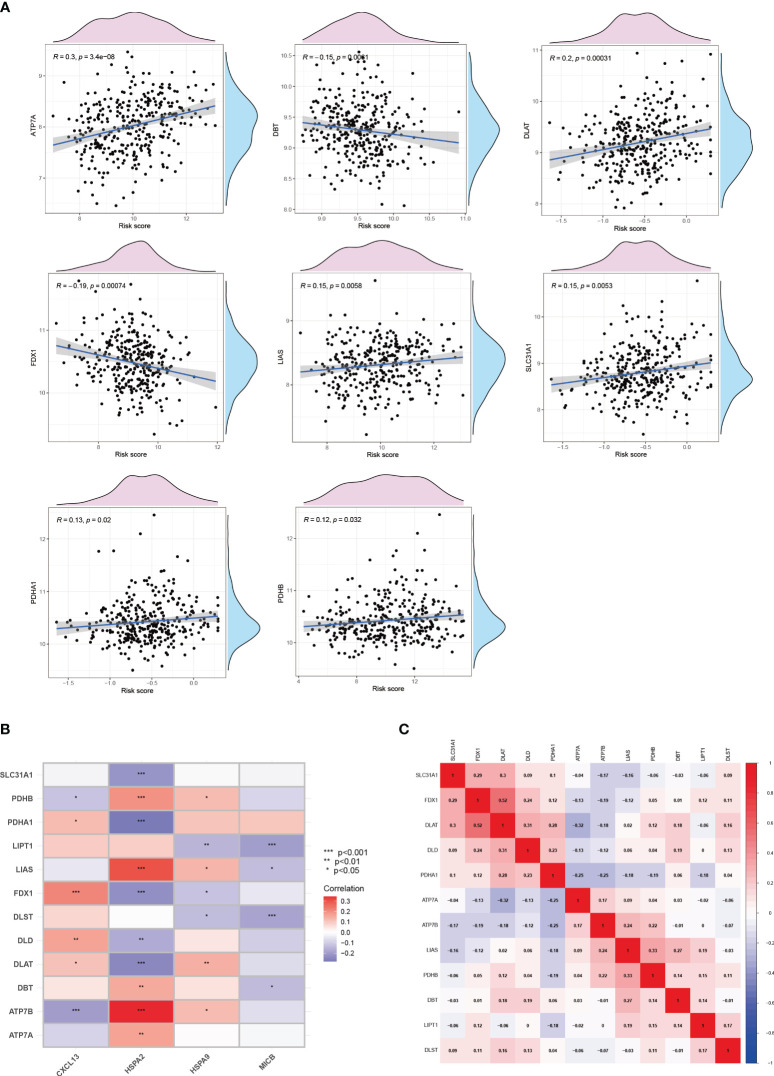
Cuproptosis and the cancer-immunity cycle. **(A)**The correlation between cuproptosis and the risk of breast cancer. **(B)** The correlation between cuproptosis and the cancer-immunity cycle. **(C)** The correlation among genes involved in cuproptosis.

Metabolic regulation of cuproptosis influences the immune processes. Furthermore, in breast cancer patients with higher risk, we explored the expression level of key cuproptosis-regulating genes (**Figure 11A**), implying that copper death serves as a prognostic biomarker for adverse outcomes in breast cancer patients, and targeting copper death may ameliorate poor prognosis. And thus, we proceeded to delve deeper into the network between cuproptosis and CICs in breast cancer patients. It is noteworthy that a significant and extensive correlation exists between Cuproptosis and CICs, indicating potential interactions between them (**Figure 11B**). In an overarching perspective, cuproptosis-related genes exhibit positive correlations with each other (**Figure 11C**).

### 3.9 Inhibition of cuproptosis-CICs impairs BC cell migration ability

**Figure 12:**
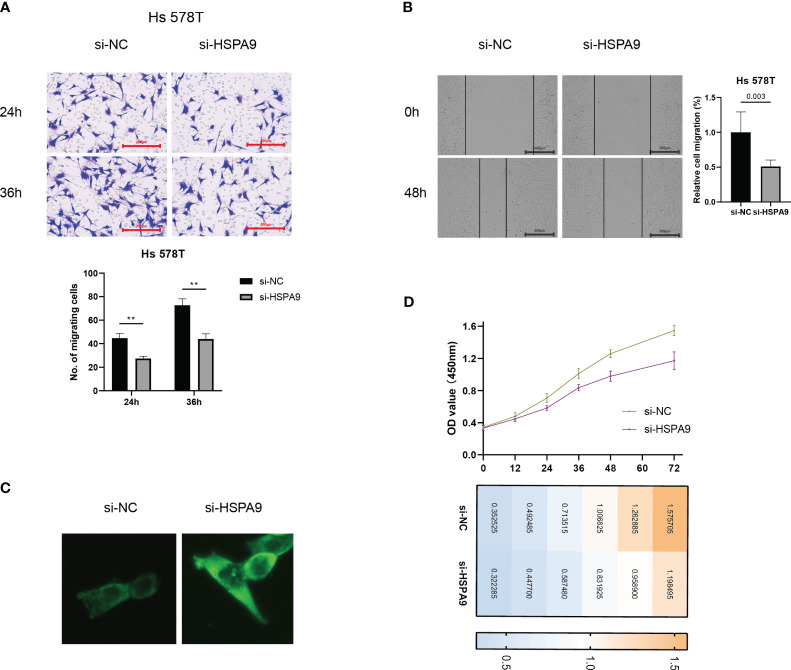
Inhibition of cuproptosis-CICs curtails migration and proliferation ability in breast cancer cells. **(A, B)** Migration capabilities of Hs 578T cells post-transfection with si-HSPA9. **(C)** Levels of Ceramide expression. **(D)** Proliferation assessment in Ts 578T cells **P < 0.01.

The metastatic propensity of breast cancer cells crucially dictates prognostic outcomes. Hence, we explored the impact of CIC activity on the migration ability of breast cancer cells. Our findings elucidated that the inhibition of HSPA9 markedly restricts the migration speed of Hs 578T cells (**Figures 12A, B**).

### 3.10 Inhibition of cuproptosis-CIC impairs proliferation in BC cells through ceramide accumulation induction

Ceramide plays a pivotal role in constraining the migration ability of tumor cells. In order to elucidate the crucial mechanisms by which cuproptosis-CICs regulate the migration of breast cancer cells, we examined the expression levels of ceramide. Following the suppression of HSPA9 expression, a significant accumulation of sphingolipids, including ceramide, was observed in breast cancer cells as compared to the control group (**Figure 12C**). Furthermore, the accumulation of ceramide exerts an impact on cellular proliferation. Hs 578T cells were transfected with siRNA and concurrent monitoring of cell viability was conducted. As depicted in **Figure 12D**, compared to the control group, the si-HSPA9 group of breast cancer cells exhibited a pronounced attenuation in proliferation over a specified time period. This suggests that HSPA9 may induce the proliferation and migration of Hs 578T cells by inhibiting the accumulation of ceramide within the cells.

## 4 Discussion

Breast cancer continues to pose significant health risks, distinguished by its diverse nature and intricate development mechanisms (35, 36). While traditional interventions like surgery, chemotherapy, and radiotherapy have notably enhanced patient survival rates, they frequently encounter challenges such as relapse and resistance. The emergence of immunotherapy has marked a promising direction in treating breast cancer, emphasizing the strategic activation of the immune system to fight the ailment (37). Nonetheless, the effectiveness of immunotherapy in this context is varied, primarily due to the heterogeneous nature of the tumor environment and the differential immune responses observed (37, 38).

Cuproptosis refers to the regulatory role of copper ions (Cu2+) in the cellular apoptosis process, both intracellularly and extracellularly. Copper, a vital trace element, is essential for sustaining life and normal cellular operations. Epigenetic alterations in appearance are intricately linked to tumor progression (39), influencing the migratory capacity of cancer cells (40), adversely affecting the prognosis and recurrence of cancer patients, and leading to drug resistance (41). Nevertheless, studies investigating the impact of epigenetic alterations in Cuproptosis-related genes on tumor progression are scarce in the literature. Recent investigations have illuminated copper’s critical role in tumor biology, particularly in modulating apoptosis. Copper can generate an oxidative stress milieu within cells, triggering apoptosis in cancer cells by elevating oxidative stress levels. This effect is facilitated by copper ions, which promote the generation of deleterious free radicals, thereby damaging the DNA, proteins, and lipids of tumor cells and initiating apoptosis. Additionally, copper can influence the functionality of the immune system, altering immune responses within the tumor microenvironment. This alteration may make tumor cells more susceptible to detection and destruction by the immune system. The concept of cuproptosis-cancer-immunity cycles (CICs) is integral in understanding the interplay between cancer cells and the immune system (42). Our research highlights the pivotal role of cuproptosis-CICs in breast cancer management, emphasizing their potential in transcending existing treatment limitations and fostering the development of personalized therapeutic approaches.

Immunotherapy has evolved into a fundamental therapeutic strategy for addressing cancer and has undergone comprehensive scrutiny (34, 43–45). This methodology encompasses the utilization of the immune system to discern and obliterate malignant cells. In this study, we conducted an extensive examination of cuproptosis-CICs within the context of breast cancer, uncovering pivotal insights into their influence on modulating immune reactions and affecting patient longevity. By categorizing breast cancer individuals based on their cuproptosis-CIC expression profiles, significant associations with survival rates and the presence of immune cells were identified (**Figure 5E**). This classification approach offers a novel perspective for understanding the inherent diversity within breast cancer and tailoring treatments to individual patients. Notably, it is intriguing that high-risk breast cancer patients exhibit heightened sensitivity to PD-1 therapy and display better prognoses compared to their low-risk counterparts (**Figure 8**). This suggests a potential overexpression of immune checkpoint markers in high-risk breast cancer patients. Through single-cell analysis, we have uncovered a connection between breast cancer cells and PD-1-expressing CD8 cells, further elucidating the mechanisms underlying the heightened sensitivity of high-risk breast cancer patients to PD-1 therapy (**Figure 7E**). Therefore, breast cancer cells likely evade T cell attacks through an immunosuppressive microenvironment, thereby driving breast cancer progression. The high expression of PD-1 in CD8 T cells among breast cancer patients with elevated cuproptosis-CIC expression further underscores the potential benefits of anti-PD-1 therapy for high-risk patients. In conclusion, our research lays the groundwork for further investigations into the functional roles of cuproptosis-CICs, which could potentially transform the methods employed in breast cancer treatment and improve the effectiveness of immunotherapies.

Ceramide, a bioactive lipid, plays a pivotal role in cellular signal transduction pathways (46). It is considered a molecule that promotes apoptosis by activating various apoptotic pathways. Our previous analysis has revealed a significant downregulation of apoptotic signaling pathways in high-risk breast cancer patients (**Figure 3F**). Furthermore, ceramide exerts a significant influence on tumor cell motility, proliferation, and immune evasion within the tumor microenvironment (47–49). Ceramide can alter cell motility by affecting the cytoskeleton of tumor cells (50), potentially impacting the polymerization of actin and thereby influencing cell migration and invasiveness. Notably, in certain cancer types, the accumulation of ceramide is associated with reduced tumor cell migration, suggesting its role in limiting cancer cell dissemination (51). Our research findings also observed poor prognosis in high-risk breast cancer patients (**Figure 5E**) and an associated state of immune suppression (**Figures 9C, G**). Therefore, we hypothesize that HSPA9 may inhibit ceramide formation, subsequently promoting breast cancer cell motility and resistance to immune therapies. This hypothesis is supported by immunofluorescence results confirming the inhibitory effect of HSPA on ceramide through siRNA-mediated HSPA9 knockdown (**Figure 12C**). This discovery further elucidates how the high expression of cuproptosis-CICs genes in breast cancer patients may lead to a poor prognosis by upregulating HSPA9, thereby shaping an immune-suppressive microenvironment and enhancing distant metastasis of breast cancer cells.

Immune suppression within the tumor microenvironment is a critical factor in cancer progression (52). Through single-cell analysis, we observed extensive crosstalk between tumor cells and macrophages in high-risk breast cancer patients (**Figure 7E**). The infiltration of macrophages significantly impacts tumor cell growth and the efficacy of immunotherapy (53). Immune infiltration analysis indicates a significant increase in M2-type macrophage infiltration in high-risk breast cancer patients (*P* < 0.001) (**Figure 9C**). Therefore, M2-type macrophage infiltration may play a crucial role in the adverse prognosis of breast cancer. Interestingly, there is a connection between breast cancer cells and PD-1-expressing CD8 T cells, and CD8 T cell infiltration is reduced in high-risk breast cancer patients. This suggests that breast cancer cells evade recognition by CD8 T cells by modulating the expression of immune checkpoints, leading to immune escape. Infiltration levels of CD4 memory T cells significantly decrease with increasing risk scores in breast cancer patients (**Figure 9B**). Thus, the dysfunction of various immune cells is a major characteristic of the breast cancer tumor microenvironment, and improving the functionality of immune cells will be a crucial strategy in breast cancer therapy.

HSPA9 makes the most significant contribution to the prognosis model, yet its study in conjunction with ceramide is scarce. By interfering with HSPA9 expression levels, the results show a significant increase in ceramide expression (**Figure 12C**). This suggests that HSPA9 may reshape the immune-suppressive microenvironment to evade recognition by CD8 T cells by inhibiting ceramide production. Additionally, HSPA9 downregulates ceramide expression, weakening ceramide’s inhibitory effect on tumor cell migration and leading to distant metastasis of breast cancer cells. Considering the impact of ceramide on tumor cell proliferation, we conducted CCK-8 experiments to observe whether HSPA9 inhibition would slow down breast cancer cell proliferation. In line with our hypothesis, we observed a significant inhibition of breast cancer cell proliferation starting at 48 hours after HSPA9 interference (**Figure 12D**). These studies highlight the crucial role of ceramide in the influence of HSPA9 on breast cancer cell migration, proliferation, and the immune microenvironment.

In our investigation, we explore the paradoxical nature of the immune system in cancer progression, which encompasses both inhibition and facilitation of tumors. This study is specifically centered on the impact of cuproptosis-CICs within the tumor microenvironment. Analyzing the complex interplay between cuproptosis-CICs and immune cells in breast cancer, we gain an enriched perspective on tumor immune responses. Our findings suggest that by targeting particular cuproptosis-CICs, it is possible to alter the immune milieu, thereby potentially shifting the balance in favor of effective antitumor immunity. This dimension of our study opens promising pathways for the creation of novel immunotherapy approaches. Such strategies could potentially increase the efficacy of breast cancer treatments through immunotherapy, leading to more favorable therapeutic results.

This investigation represents a major advance in comprehending the complex interplay between breast cancer and the immune system. Our work illuminates the predictive and prognostic significance of cuproptosis-CICs, opening new paths for enhancing breast cancer treatments. Acknowledging our study’s constraints, such as the necessity for broader and more varied participant groups to corroborate our conclusions, is crucial. It’s imperative for subsequent studies to extend the clinical relevance of our findings, delve into the therapeutic implications of selectively targeting cuproptosis-CICs, and incorporate these insights into innovative treatment frameworks. Such efforts are essential not only for deepening our grasp of breast cancer immunology but also for spurring the development of more tailored and efficacious therapeutic approaches.

## 5 Conclusion

In this research, we delve into the complexities of the cuproptosis-immune interplay in breast cancer, shedding light on novel prospects for creating specialized immunotherapies. This approach is primarily directed at enhancing the efficacy of breast cancer treatments, thereby potentially elevating patient prognosis.

## Data availability statement

The original contributions presented in the study are included in the article/**Supplementary Material**. Further inquiries can be directed to the corresponding author.

## Ethics statement

Ethical approval was not required for the studies on humans in accordance with the local legislation and institutional requirements because only commercially available established cell lines were used.

## Author contributions

XL: Data curation, Formal Analysis, Investigation, Methodology, Software, Visualization, Writing – original draft. FX: Data curation, Formal Analysis, Investigation, Software, Visualization, Writing – original draft. KZ: Formal Analysis, Methodology, Writing – original draft. YL: Investigation, Resources, Writing – review & editing. GY: Investigation, Resources, Writing – review & editing. XZ: Investigation, Resources, Writing – review & editing. YQ: Conceptualization, Funding acquisition, Investigation, Project administration, Supervision, Validation, Writing – review & editing.
